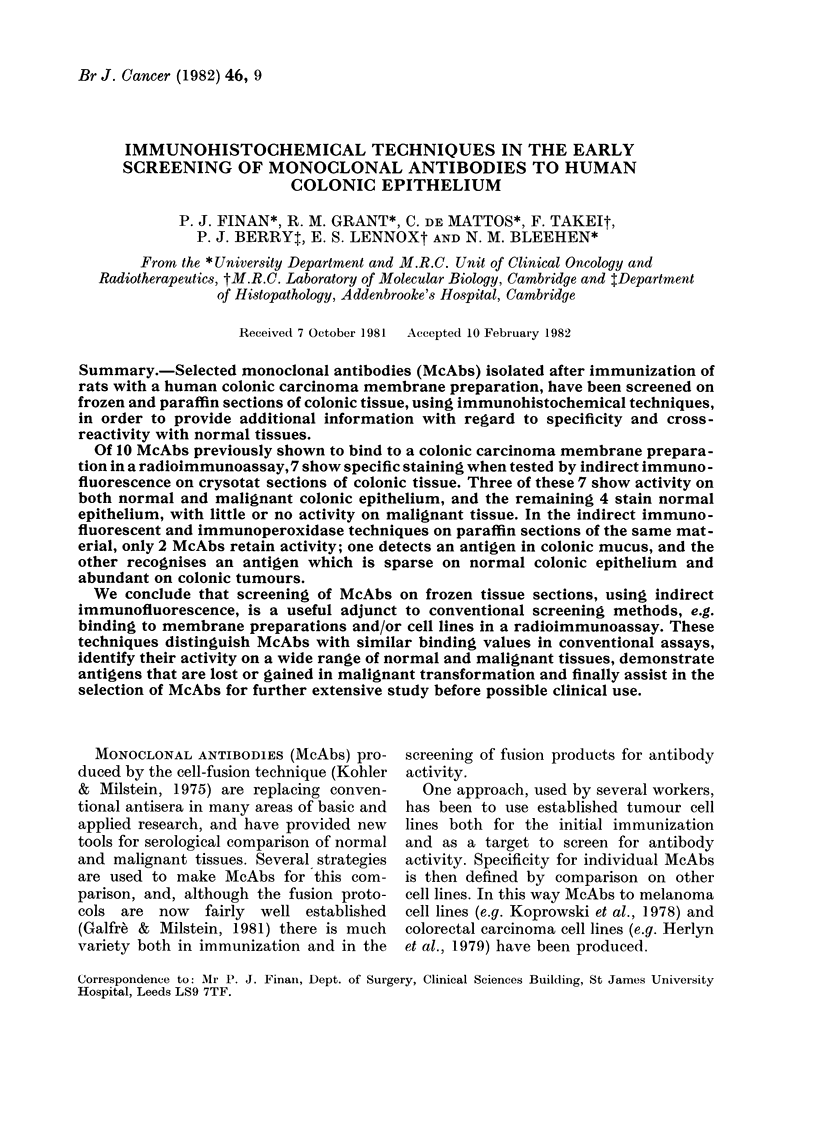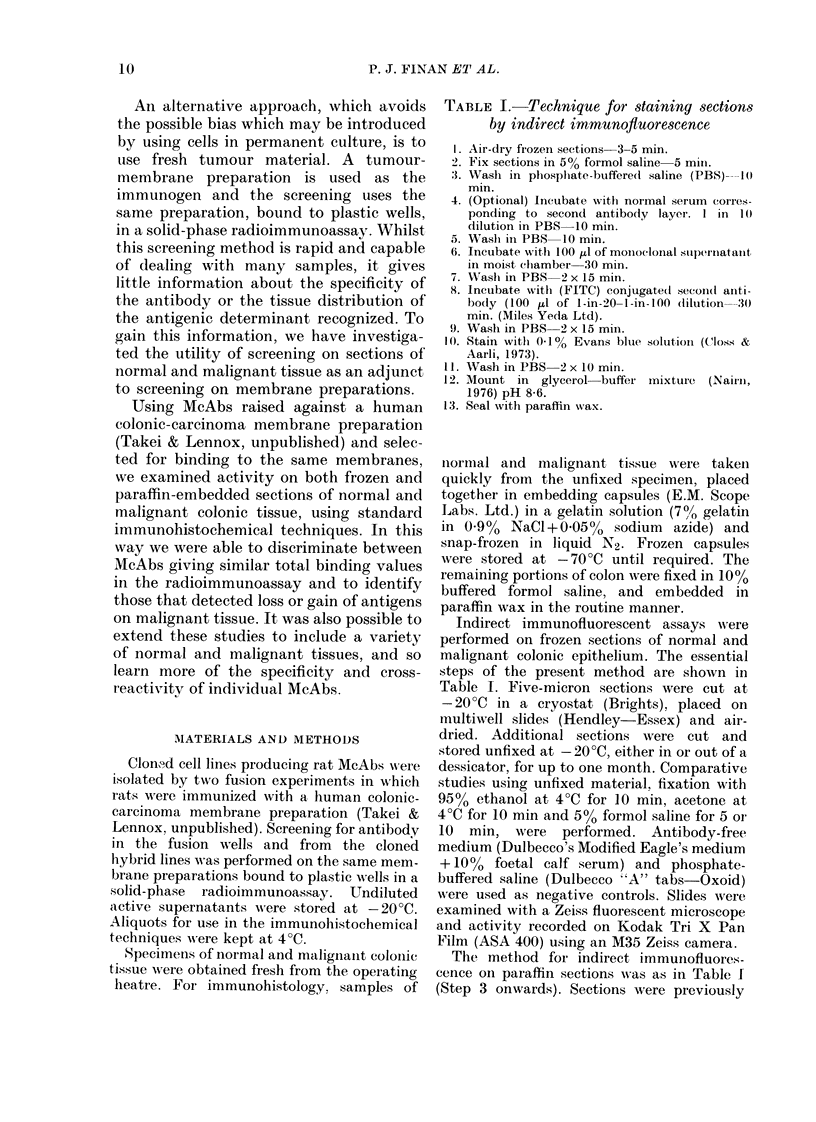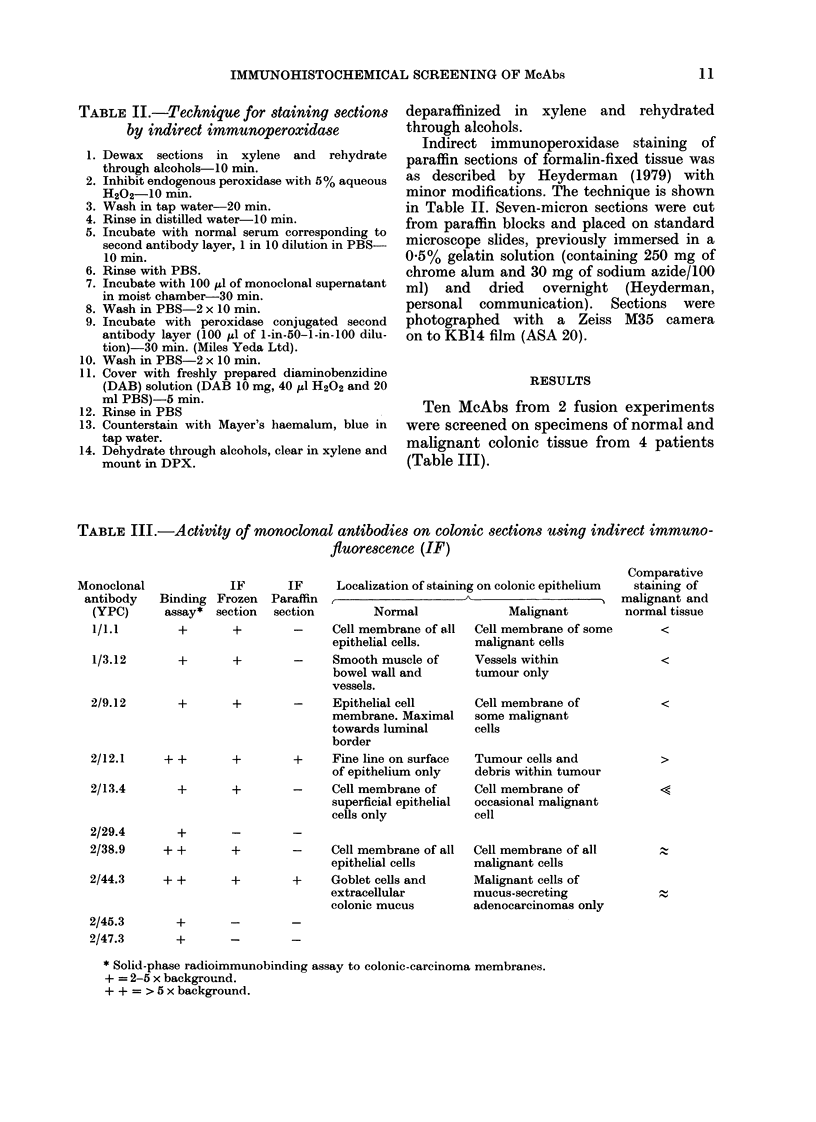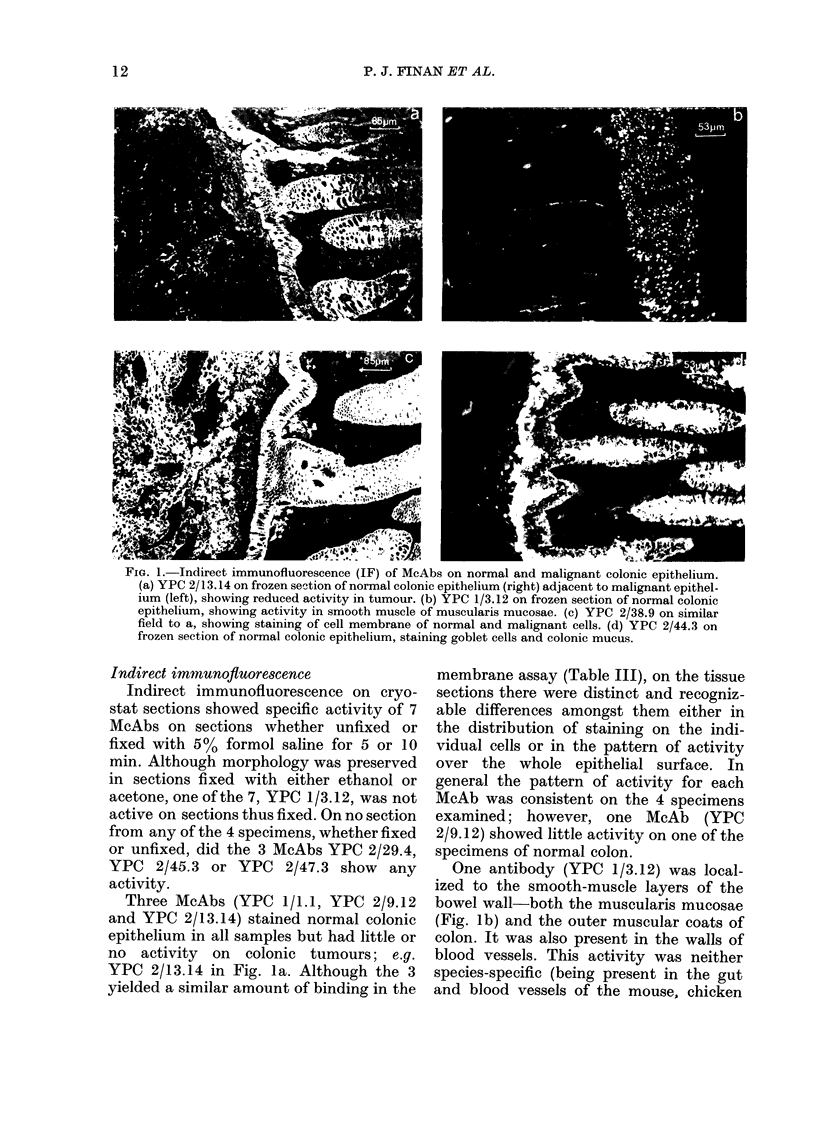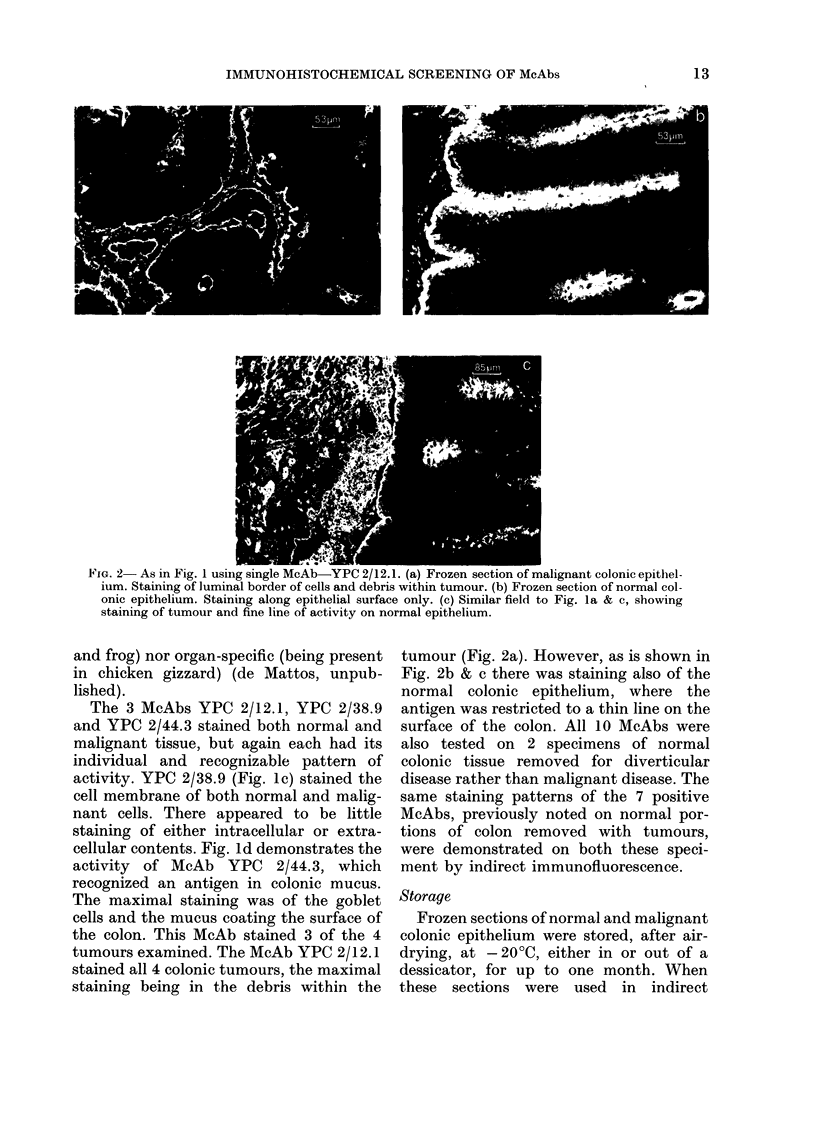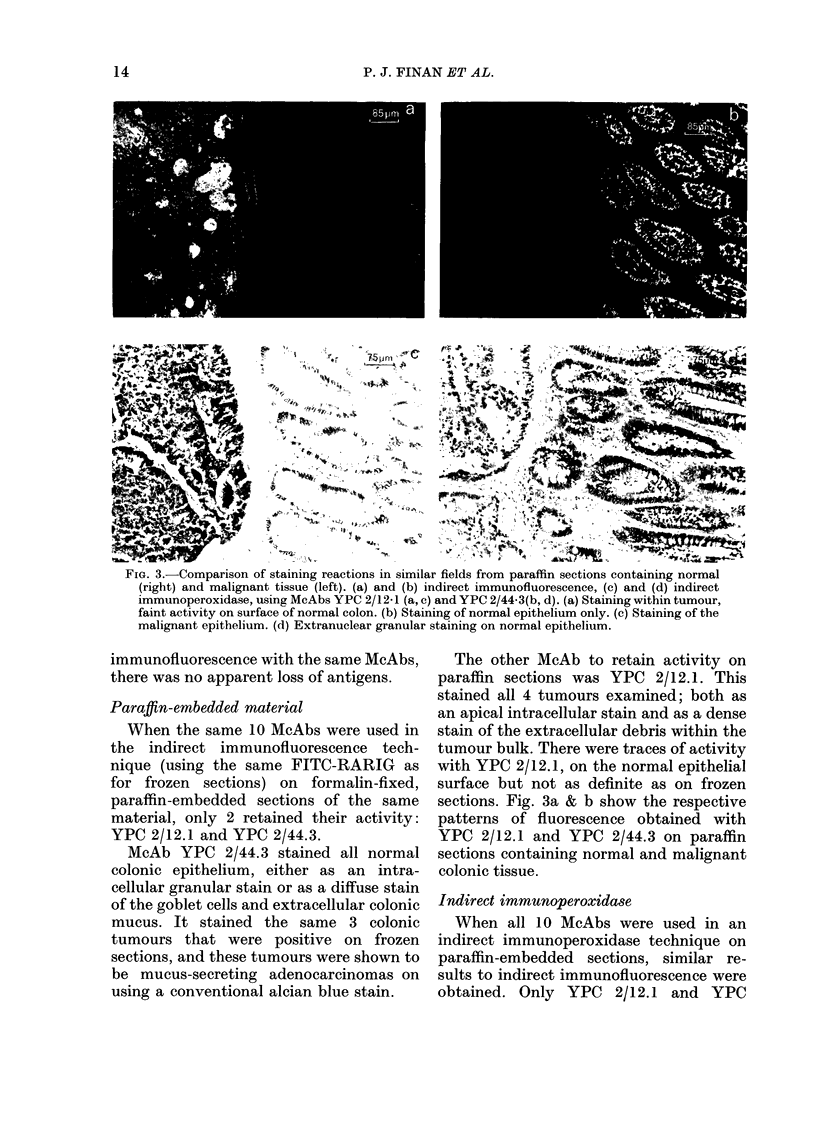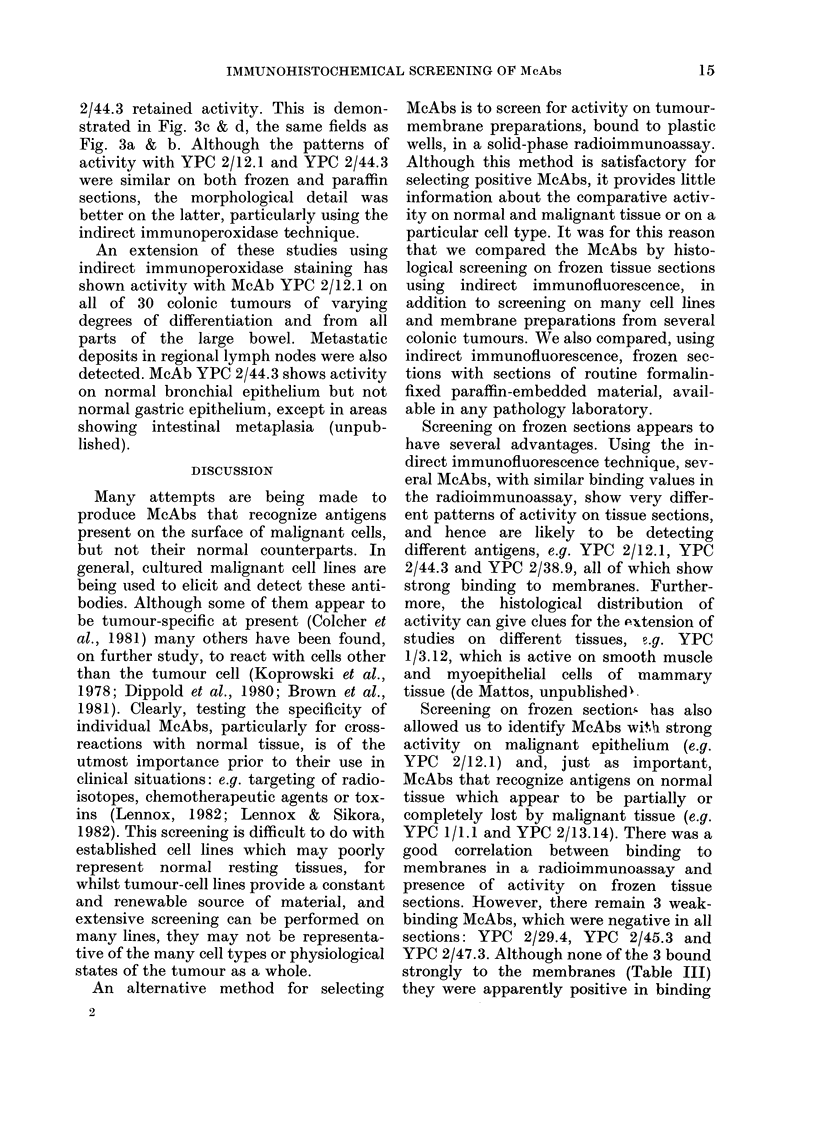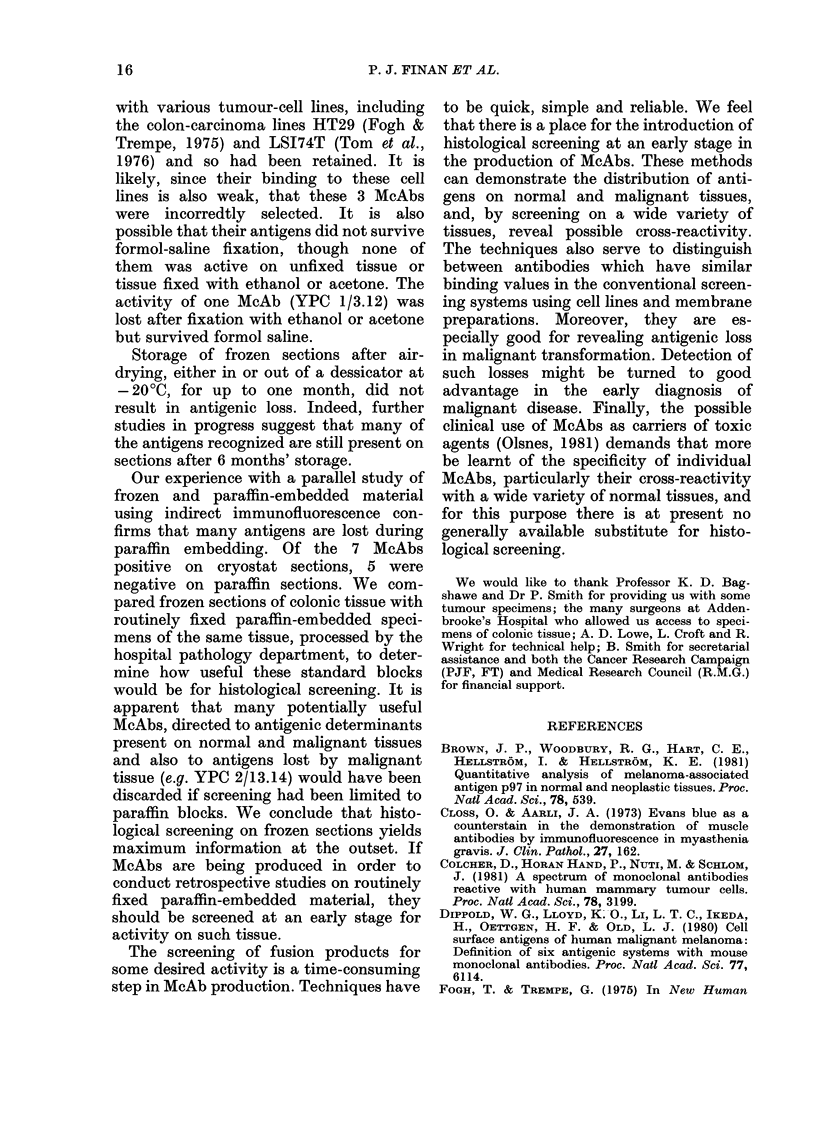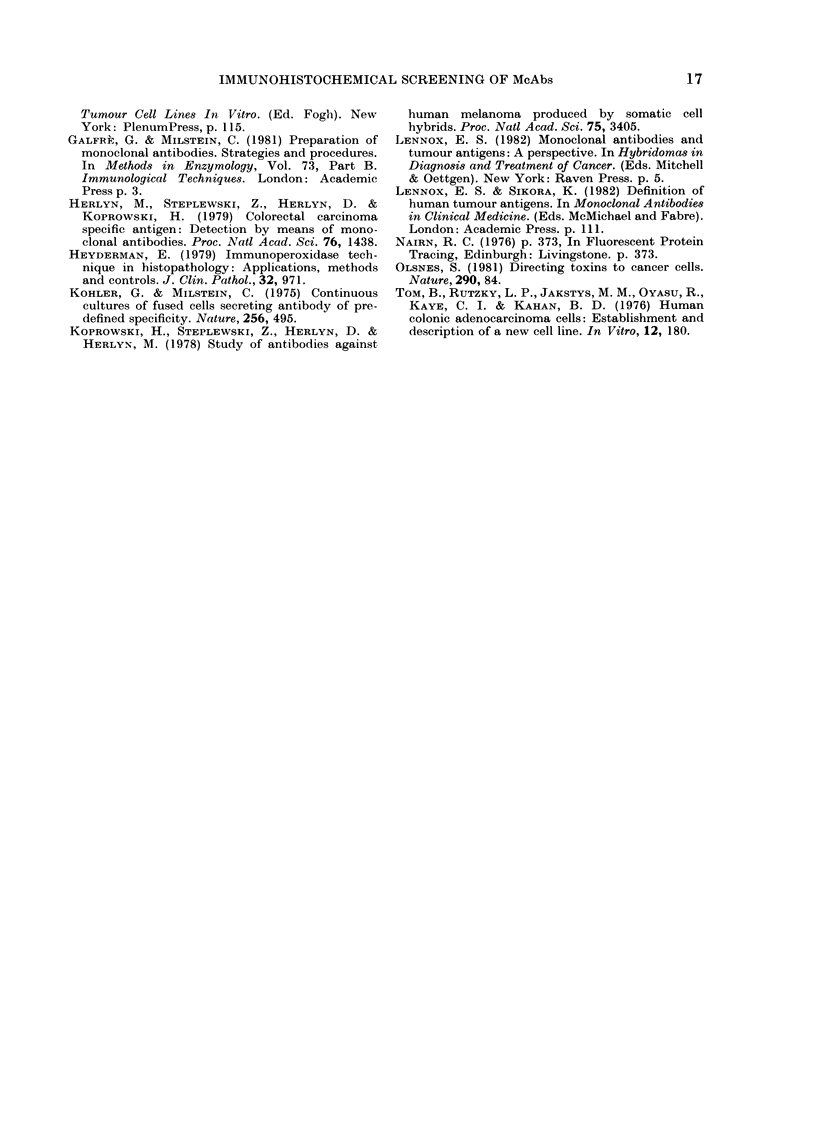# Immunohistochemical techniques in the early screening of monoclonal antibodies to human colonic epithelium

**DOI:** 10.1038/bjc.1982.158

**Published:** 1982-07

**Authors:** P. J. Finan, R. M. Grant, C. de Mattos, F. Takei, P. J. Berry, E. S. Lennox, N. M. Bleehen

## Abstract

**Images:**


*Br J. Cancer* (1982) **46**, 9

### IMMUNOHISTOCHEMICAL TECHNIQUES IN THE EARLY SCREENING OF MONOCLONAL ANTIBODIES TO HUMAN COLONIC EPITHELIUM

P. J. FINAN*, R. M. GRANT*, C. DE MATTOS*, F. TAKEI†, P. J. BERRY‡, E. S. LENNOX† AND N. M. BLEEHEN*

*From the *University Department and M.R.C. Unit of Clinical Oncology and Radiotherapeutics, †M.R.C. Laboratory of Molecular Biology, Cambridge and ‡Department of Histopathology, Addenbrooke's Hospital, Cambridge*

Received 7 October 1981 Accepted 10 February 1982

**Summary.—Selected monoclonal antibodies (McAbs) isolated after immunization of rats with a human colonic carcinoma membrane preparation, have been screened on frozen and paraffin sections of colonic tissue, using immunohistochemical techniques, in order to provide additional information with regard to specificity and cross-reactivity with normal tissues.**

**Of 10 McAbs previously shown to bind to a colonic carcinoma membrane preparation in a radioimmunoassay, 7 show specific staining when tested by indirect immunofluorescence on crysotat sections of colonic tissue. Three of these 7 show activity on both normal and malignant colonic epithelium, and the remaining 4 stain normal epithelium, with little or no activity on malignant tissue. In the indirect immunofluorescent and immunoperoxidase techniques on paraffin sections of the same material, only 2 McAbs retain activity; one detects an antigen in colonic mucus, and the other recognises an antigen which is sparse on normal colonic epithelium and abundant on colonic tumours.**

**We conclude that screening of McAbs on frozen tissue sections, using indirect immunofluorescence, is a useful adjunct to conventional screening methods, *e.g.* binding to membrane preparations and/or cell lines in a radioimmunoassay. These techniques distinguish McAbs with similar binding values in conventional assays, identify their activity on a wide range of normal and malignant tissues, demonstrate antigens that are lost or gained in malignant transformation and finally assist in the selection of McAbs for further extensive study before possible clinical use.**

MONOCLONAL ANTIBODIES (McAbs) produced by the cell-fusion technique (Kohler & Milstein, 1975) are replacing conventional antisera in many areas of basic and applied research, and have provided new tools for serological comparison of normal and malignant tissues. Several strategies are used to make McAbs for this comparison, and, although the fusion protocols are now fairly well established (Galfrè & Milstein, 1981) there is much variety both in immunization and in the screening of fusion products for antibody activity.

One approach, used by several workers, has been to use established tumour cell lines both for the initial immunization and as a target to screen for antibody activity. Specificity for individual McAbs is then defined by comparison on other cell lines. In this way McAbs to melanoma cell lines (*e.g.* Koprowski *et al.*, 1978) and colorectal carcinoma cell lines (*e.g.* Herlyn *et al.*, 1979) have been produced.

Correspondence to: Mr P. J. Finan, Dept. of Surgery, Clinical Sciences Building, St James University Hospital, Leeds LS9 7TF.

An alternative approach, which avoids the possible bias which may be introduced by using cells in permanent culture, is to use fresh tumour material. A tumour-membrane preparation is used as the immunogen and the screening uses the same preparation, bound to plastic wells, in a solid-phase radioimmunoassay. Whilst this screening method is rapid and capable of dealing with many samples, it gives little information about the specificity of the antibody or the tissue distribution of the antigenic determinant recognized. To gain this information, we have investigated the utility of screening on sections of normal and malignant tissue as an adjunct to screening on membrane preparations.

Using McAbs raised against a human colonic-carcinoma membrane preparation (Takei & Lennox, unpublished) and selected for binding to the same membranes, we examined activity on both frozen and paraffin-embedded sections of normal and malignant colonic tissue, using standard immunohistochemical techniques. In this way we were able to discriminate between McAbs giving similar total binding values in the radioimmunoassay and to identify those that detected loss or gain of antigens on malignant tissue. It was also possible to extend these studies to include a variety of normal and malignant tissues, and so learn more of the specificity and cross-reactivity of individual McAbs.

### MATERIALS AND METHODS

Cloned cell lines producing rat McAbs were isolated by two fusion experiments in which rats were immunized with a human colonic-carcinoma membrane preparation (Takei & Lennox, unpublished). Screening for antibody in the fusion wells and from the cloned hybrid lines was performed on the same membrane preparations bound to plastic wells in a solid-phase radioimmunoassay. Undiluted active supernatants were stored at $-20°C$. Aliquots for use in the immunohistochemical techniques were kept at 4°C.

Specimens of normal and malignant colonic tissue were obtained fresh from the operating heatre. For immunohistology, samples of normal and malignant tissue were taken quickly from the unfixed specimen, placed together in embedding capsules (E.M. Scope Labs. Ltd.) in a gelatin solution (7% gelatin in 0·9% NaCl+0·05% sodium azide) and snap-frozen in liquid $N_2$. Frozen capsules were stored at $-70°C$ until required. The remaining portions of colon were fixed in 10% buffered formol saline, and embedded in paraffin wax in the routine manner.

TABLE I.—*Technique for staining sections by indirect immunofluorescence*

1. Air-dry frozen sections—3–5 min.
2. Fix sections in 5% formol saline—5 min.
3. Wash in phosphate-buffered saline (PBS)—10 min.
4. (Optional) Incubate with normal serum corresponding to second antibody layer. 1 in 10 dilution in PBS—10 min.
5. Wash in PBS—10 min.
6. Incubate with 100 μl of monoclonal supernatant in moist chamber—30 min.
7. Wash in PBS—2 x 15 min.
8. Incubate with (FITC) conjugated second antibody (100 μl of 1-in-20–1-in-100 dilution—30 min. (Miles Yeda Ltd).
9. Wash in PBS—2 x 15 min.
10. Stain with 0·1% Evans blue solution (Closs & Aarli, 1973).
11. Wash in PBS—2 x 10 min.
12. Mount in glycerol—buffer mixture (Nairn, 1976) pH 8·6.
13. Seal with paraffin wax.

Indirect immunofluorescent assays were performed on frozen sections of normal and malignant colonic epithelium. The essential steps of the present method are shown in Table I. Five-micron sections were cut at $-20°C$ in a cryostat (Brights), placed on multiwell slides (Hendley—Essex) and air-dried. Additional sections were cut and stored unfixed at $-20°C$, either in or out of a dessicator, for up to one month. Comparative studies using unfixed material, fixation with 95% ethanol at 4°C for 10 min, acetone at 4°C for 10 min and 5% formol saline for 5 or 10 min, were performed. Antibody-free medium (Dulbecco's Modified Eagle's medium +10% foetal calf serum) and phosphate-buffered saline (Dulbecco "A" tabs—Oxoid) were used as negative controls. Slides were examined with a Zeiss fluorescent microscope and activity recorded on Kodak Tri X Pan Film (ASA 400) using an M35 Zeiss camera.

The method for indirect immunofluorescence on paraffin sections was as in Table I (Step 3 onwards). Sections were previously

TABLE II.—*Technique for staining sections by indirect immunoperoxidase*

1. Dewax sections in xylene and rehydrate through alcohols—10 min.
2. Inhibit endogenous peroxidase with 5% aqueous $H_2O_2$—10 min.
3. Wash in tap water—20 min.
4. Rinse in distilled water—10 min.
5. Incubate with normal serum corresponding to second antibody layer, 1 in 10 dilution in PBS—10 min.
6. Rinse with PBS.
7. Incubate with 100 μl of monoclonal supernatant in moist chamber—30 min.
8. Wash in PBS—2 x 10 min.
9. Incubate with peroxidase conjugated second antibody layer (100 μl of 1-in-50–1-in-100 dilution)—30 min. (Miles Yeda Ltd).
10. Wash in PBS—2 x 10 min.
11. Cover with freshly prepared diaminobenzidine (DAB) solution (DAB 10 mg, 40 μl $H_2O_2$ and 20 ml PBS)—5 min.
12. Rinse in PBS
13. Counterstain with Mayer's haemalum, blue in tap water.
14. Dehydrate through alcohols, clear in xylene and mount in DPX.

deparaffinized in xylene and rehydrated through alcohols.

Indirect immunoperoxidase staining of paraffin sections of formalin-fixed tissue was as described by Heyderman (1979) with minor modifications. The technique is shown in Table II. Seven-micron sections were cut from paraffin blocks and placed on standard microscope slides, previously immersed in a 0·5% gelatin solution (containing 250 mg of chrome alum and 30 mg of sodium azide/100 ml) and dried overnight (Heyderman, personal communication). Sections were photographed with a Zeiss M35 camera on to KB14 film (ASA 20).

### RESULTS

Ten McAbs from 2 fusion experiments were screened on specimens of normal and malignant colonic tissue from 4 patients (Table III).

TABLE III.—*Activity of monoclonal antibodies on colonic sections using indirect immunofluorescence (IF)*

| Monoclonal antibody (YPC) | Binding assay* | IF Frozen section | IF Paraffin section | Localization of staining on colonic epithelium: Normal | Malignant | Comparative staining of malignant and normal tissue |
|---|---|---|---|---|---|---|
| 1/1.1 | + | + | − | Cell membrane of all epithelial cells. | Cell membrane of some malignant cells | < |
| 1/3.12 | + | + | − | Smooth muscle of bowel wall and vessels. | Vessels within tumour only | < |
| 2/9.12 | + | + | − | Epithelial cell membrane. Maximal towards luminal border | Cell membrane of some malignant cells | < |
| 2/12.1 | ++ | + | + | Fine line on surface of epithelium only | Tumour cells and debris within tumour | > |
| 2/13.4 | + | + | − | Cell membrane of superficial epithelial cells only | Cell membrane of occasional malignant cell | ⩽ |
| 2/29.4 | + | − | − | | | |
| 2/38.9 | ++ | + | − | Cell membrane of all epithelial cells | Cell membrane of all malignant cells | ≈ |
| 2/44.3 | ++ | + | + | Goblet cells and extracellular colonic mucus | Malignant cells of mucus-secreting adenocarcinomas only | ≈ |
| 2/45.3 | + | − | − | | | |
| 2/47.3 | + | − | − | | | |

* Solid-phase radioimmunobinding assay to colonic-carcinoma membranes.
+ = 2–5 x background.
+ + = > 5 x background.

FIG. 1.—Indirect immunofluorescence (IF) of McAbs on normal and malignant colonic epithelium. (a) YPC 2/13.14 on frozen section of normal colonic epithelium (right) adjacent to malignant epithelium (left), showing reduced activity in tumour. (b) YPC 1/3.12 on frozen section of normal colonic epithelium, showing activity in smooth muscle of muscularis mucosae. (c) YPC 2/38.9 on similar field to a, showing staining of cell membrane of normal and malignant cells. (d) YPC 2/44.3 on frozen section of normal colonic epithelium, staining goblet cells and colonic mucus.

*Indirect immunofluorescence*

Indirect immunofluorescence on cryostat sections showed specific activity of 7 McAbs on sections whether unfixed or fixed with 5% formol saline for 5 or 10 min. Although morphology was preserved in sections fixed with either ethanol or acetone, one of the 7, YPC 1/3.12, was not active on sections thus fixed. On no section from any of the 4 specimens, whether fixed or unfixed, did the 3 McAbs YPC 2/29.4, YPC 2/45.3 or YPC 2/47.3 show any activity.

Three McAbs (YPC 1/1.1, YPC 2/9.12 and YPC 2/13.14) stained normal colonic epithelium in all samples but had little or no activity on colonic tumours; *e.g.* YPC 2/13.14 in Fig. 1a. Although the 3 yielded a similar amount of binding in the membrane assay (Table III), on the tissue sections there were distinct and recognizable differences amongst them either in the distribution of staining on the individual cells or in the pattern of activity over the whole epithelial surface. In general the pattern of activity for each McAb was consistent on the 4 specimens examined; however, one McAb (YPC 2/9.12) showed little activity on one of the specimens of normal colon.

One antibody (YPC 1/3.12) was localized to the smooth-muscle layers of the bowel wall—both the muscularis mucosae (Fig. 1b) and the outer muscular coats of colon. It was also present in the walls of blood vessels. This activity was neither species-specific (being present in the gut and blood vessels of the mouse, chicken

FIG. 2— As in Fig. 1 using single McAb—YPC 2/12.1. (a) Frozen section of malignant colonic epithelium. Staining of luminal border of cells and debris within tumour. (b) Frozen section of normal colonic epithelium. Staining along epithelial surface only. (c) Similar field to Fig. 1a & c, showing staining of tumour and fine line of activity on normal epithelium.

and frog) nor organ-specific (being present in chicken gizzard) (de Mattos, unpublished).

The 3 McAbs YPC 2/12.1, YPC 2/38.9 and YPC 2/44.3 stained both normal and malignant tissue, but again each had its individual and recognizable pattern of activity. YPC 2/38.9 (Fig. 1c) stained the cell membrane of both normal and malignant cells. There appeared to be little staining of either intracellular or extracellular contents. Fig. 1d demonstrates the activity of McAb YPC 2/44.3, which recognized an antigen in colonic mucus. The maximal staining was of the goblet cells and the mucus coating the surface of the colon. This McAb stained 3 of the 4 tumours examined. The McAb YPC 2/12.1 stained all 4 colonic tumours, the maximal staining being in the debris within the tumour (Fig. 2a). However, as is shown in Fig. 2b & c there was staining also of the normal colonic epithelium, where the antigen was restricted to a thin line on the surface of the colon. All 10 McAbs were also tested on 2 specimens of normal colonic tissue removed for diverticular disease rather than malignant disease. The same staining patterns of the 7 positive McAbs, previously noted on normal portions of colon removed with tumours, were demonstrated on both these speciment by indirect immunofluorescence.

*Storage*

Frozen sections of normal and malignant colonic epithelium were stored, after air-drying, at $-20°C$, either in or out of a dessicator, for up to one month. When these sections were used in indirect

FIG. 3.—Comparison of staining reactions in similar fields from paraffin sections containing normal (right) and malignant tissue (left). (a) and (b) indirect immunofluorescence, (c) and (d) indirect immunoperoxidase, using McAbs YPC 2/12·1 (a, c) and YPC 2/44·3(b, d). (a) Staining within tumour, faint activity on surface of normal colon. (b) Staining of normal epithelium only. (c) Staining of the malignant epithelium. (d) Extranuclear granular staining on normal epithelium.

immunofluorescence with the same McAbs, there was no apparent loss of antigens.

*Paraffin-embedded material*

When the same 10 McAbs were used in the indirect immunofluorescence technique (using the same FITC-RARIG as for frozen sections) on formalin-fixed, paraffin-embedded sections of the same material, only 2 retained their activity: YPC 2/12.1 and YPC 2/44.3.

McAb YPC 2/44.3 stained all normal colonic epithelium, either as an intracellular granular stain or as a diffuse stain of the goblet cells and extracellular colonic mucus. It stained the same 3 colonic tumours that were positive on frozen sections, and these tumours were shown to be mucus-secreting adenocarcinomas on using a conventional alcian blue stain.

The other McAb to retain activity on paraffin sections was YPC 2/12.1. This stained all 4 tumours examined; both as an apical intracellular stain and as a dense stain of the extracellular debris within the tumour bulk. There were traces of activity with YPC 2/12.1, on the normal epithelial surface but not as definite as on frozen sections. Fig. 3a & b show the respective patterns of fluorescence obtained with YPC 2/12.1 and YPC 2/44.3 on paraffin sections containing normal and malignant colonic tissue.

*Indirect immunoperoxidase*

When all 10 McAbs were used in an indirect immunoperoxidase technique on paraffin-embedded sections, similar results to indirect immunofluorescence were obtained. Only YPC 2/12.1 and YPC

2/44.3 retained activity. This is demonstrated in Fig. 3c & d, the same fields as Fig. 3a & b. Although the patterns of activity with YPC 2/12.1 and YPC 2/44.3 were similar on both frozen and paraffin sections, the morphological detail was better on the latter, particularly using the indirect immunoperoxidase technique.

An extension of these studies using indirect immunoperoxidase staining has shown activity with McAb YPC 2/12.1 on all of 30 colonic tumours of varying degrees of differentiation and from all parts of the large bowel. Metastatic deposits in regional lymph nodes were also detected. McAb YPC 2/44.3 shows activity on normal bronchial epithelium but not normal gastric epithelium, except in areas showing intestinal metaplasia (unpublished).

### DISCUSSION

Many attempts are being made to produce McAbs that recognize antigens present on the surface of malignant cells, but not their normal counterparts. In general, cultured malignant cell lines are being used to elicit and detect these antibodies. Although some of them appear to be tumour-specific at present (Colcher *et al.*, 1981) many others have been found, on further study, to react with cells other than the tumour cell (Koprowski *et al.*, 1978; Dippold *et al.*, 1980; Brown *et al.*, 1981). Clearly, testing the specificity of individual McAbs, particularly for cross-reactions with normal tissue, is of the utmost importance prior to their use in clinical situations: *e.g.* targeting of radioisotopes, chemotherapeutic agents or toxins (Lennox, 1982; Lennox & Sikora, 1982). This screening is difficult to do with established cell lines which may poorly represent normal resting tissues, for whilst tumour-cell lines provide a constant and renewable source of material, and extensive screening can be performed on many lines, they may not be representative of the many cell types or physiological states of the tumour as a whole.

An alternative method for selecting McAbs is to screen for activity on tumour-membrane preparations, bound to plastic wells, in a solid-phase radioimmunoassay. Although this method is satisfactory for selecting positive McAbs, it provides little information about the comparative activity on normal and malignant tissue or on a particular cell type. It was for this reason that we compared the McAbs by histological screening on frozen tissue sections using indirect immunofluorescence, in addition to screening on many cell lines and membrane preparations from several colonic tumours. We also compared, using indirect immunofluorescence, frozen sections with sections of routine formalin-fixed paraffin-embedded material, available in any pathology laboratory.

Screening on frozen sections appears to have several advantages. Using the indirect immunofluorescence technique, several McAbs, with similar binding values in the radioimmunoassay, show very different patterns of activity on tissue sections, and hence are likely to be detecting different antigens, *e.g.* YPC 2/12.1, YPC 2/44.3 and YPC 2/38.9, all of which show strong binding to membranes. Furthermore, the histological distribution of activity can give clues for the extension of studies on different tissues, *e.g.* YPC 1/3.12, which is active on smooth muscle and myoepithelial cells of mammary tissue (de Mattos, unpublished).

Screening on frozen sections has also allowed us to identify McAbs with strong activity on malignant epithelium (*e.g.* YPC 2/12.1) and, just as important, McAbs that recognize antigens on normal tissue which appear to be partially or completely lost by malignant tissue (*e.g.* YPC 1/1.1 and YPC 2/13.14). There was a good correlation between binding to membranes in a radioimmunoassay and presence of activity on frozen tissue sections. However, there remain 3 weak-binding McAbs, which were negative in all sections: YPC 2/29.4, YPC 2/45.3 and YPC 2/47.3. Although none of the 3 bound strongly to the membranes (Table III) they were apparently positive in binding with various tumour-cell lines, including the colon-carcinoma lines HT29 (Fogh & Trempe, 1975) and LSI74T (Tom *et al.*, 1976) and so had been retained. It is likely, since their binding to these cell lines is also weak, that these 3 McAbs were incorredtly selected. It is also possible that their antigens did not survive formol-saline fixation, though none of them was active on unfixed tissue or tissue fixed with ethanol or acetone. The activity of one McAb (YPC 1/3.12) was lost after fixation with ethanol or acetone but survived formol saline.

Storage of frozen sections after air-drying, either in or out of a dessicator at −20°C, for up to one month, did not result in antigenic loss. Indeed, further studies in progress suggest that many of the antigens recognized are still present on sections after 6 months' storage.

Our experience with a parallel study of frozen and paraffin-embedded material using indirect immunofluorescence confirms that many antigens are lost during paraffin embedding. Of the 7 McAbs positive on cryostat sections, 5 were negative on paraffin sections. We compared frozen sections of colonic tissue with routinely fixed paraffin-embedded specimens of the same tissue, processed by the hospital pathology department, to determine how useful these standard blocks would be for histological screening. It is apparent that many potentially useful McAbs, directed to antigenic determinants present on normal and malignant tissues and also to antigens lost by malignant tissue (*e.g.* YPC 2/13.14) would have been discarded if screening had been limited to paraffin blocks. We conclude that histological screening on frozen sections yields maximum information at the outset. If McAbs are being produced in order to conduct retrospective studies on routinely fixed paraffin-embedded material, they should be screened at an early stage for activity on such tissue.

The screening of fusion products for some desired activity is a time-consuming step in McAb production. Techniques have to be quick, simple and reliable. We feel that there is a place for the introduction of histological screening at an early stage in the production of McAbs. These methods can demonstrate the distribution of antigens on normal and malignant tissues, and, by screening on a wide variety of tissues, reveal possible cross-reactivity. The techniques also serve to distinguish between antibodies which have similar binding values in the conventional screening systems using cell lines and membrane preparations. Moreover, they are especially good for revealing antigenic loss in malignant transformation. Detection of such losses might be turned to good advantage in the early diagnosis of malignant disease. Finally, the possible clinical use of McAbs as carriers of toxic agents (Olsnes, 1981) demands that more be learnt of the specificity of individual McAbs, particularly their cross-reactivity with a wide variety of normal tissues, and for this purpose there is at present no generally available substitute for histological screening.

We would like to thank Professor K. D. Bagshawe and Dr P. Smith for providing us with some tumour specimens; the many surgeons at Addenbrooke's Hospital who allowed us access to specimens of colonic tissue; A. D. Lowe, L. Croft and R. Wright for technical help; B. Smith for secretarial assistance and both the Cancer Research Campaign (PJF, FT) and Medical Research Council (R.M.G.) for financial support.

### REFERENCES

Brown, J. P., Woodbury, R. G., Hart, C. E., Hellström, I. & Hellström, K. E. (1981) Quantitative analysis of melanoma-associated antigen p97 in normal and neoplastic tissues. *Proc. Natl Acad. Sci.*, **78**, 539.

Closs, O. & Aarli, J. A. (1973) Evans blue as a counterstain in the demonstration of muscle antibodies by immunofluorescence in myasthenia gravis. *J. Clin. Pathol.*, **27**, 162.

Colcher, D., Horan Hand, P., Nuti, M. & Schlom, J. (1981) A spectrum of monoclonal antibodies reactive with human mammary tumour cells. *Proc. Natl Acad. Sci.*, **78**, 3199.

Dippold, W. G., Lloyd, K. O., Li, L. T. C., Ikeda, H., Oettgen, H. F. & Old, L. J. (1980) Cell surface antigens of human malignant melanoma: Definition of six antigenic systems with mouse monoclonal antibodies. *Proc. Natl Acad. Sci.* **77**, 6114.

Fogh, T. & Trempe, G. (1975) In *New Human*

*Tumour Cell Lines In Vitro*. (Ed. Fogh). New York: PlenumPress, p. 115.

Galfrè, G. & Milstein, C. (1981) Preparation of monoclonal antibodies. Strategies and procedures. In *Methods in Enzymology*, Vol. 73, Part B. *Immunological Techniques*. London: Academic Press p. 3.

Herlyn, M., Steplewski, Z., Herlyn, D. & Koprowski, H. (1979) Colorectal carcinoma specific antigen: Detection by means of monoclonal antibodies. *Proc. Natl Acad. Sci.* **76**, 1438.

Heyderman, E. (1979) Immunoperoxidase technique in histopathology: Applications, methods and controls. *J. Clin. Pathol.*, **32**, 971.

Kohler, G. & Milstein, C. (1975) Continuous cultures of fused cells secreting antibody of predefined specificity. *Nature*, **256**, 495.

Koprowski, H., Steplewski, Z., Herlyn, D. & Herlyn, M. (1978) Study of antibodies against human melanoma produced by somatic cell hybrids. *Proc. Natl Acad. Sci.* **75**, 3405.

Lennox, E. S. (1982) Monoclonal antibodies and tumour antigens: A perspective. In *Hybridomas in Diagnosis and Treatment of Cancer*. (Eds. Mitchell & Oettgen). New York: Raven Press. p. 5.

Lennox, E. S. & Sikora, K. (1982) Definition of human tumour antigens. In *Monoclonal Antibodies in Clinical Medicine*. (Eds. McMichael and Fabre). London: Academic Press. p. 111.

Nairn, R. C. (1976) p. 373, In Fluorescent Protein Tracing, Edinburgh: Livingstone. p. 373.

Olsnes, S. (1981) Directing toxins to cancer cells. *Nature*, **290**, 84.

Tom, B., Rutzky, L. P., Jakstys, M. M., Oyasu, R., Kaye, C. I. & Kahan, B. D. (1976) Human colonic adenocarcinoma cells: Establishment and description of a new cell line. *In Vitro*, **12**, 180.